# Effect of Tai Chi alone or as additional therapy on low back pain: Systematic review and meta-analysis of randomized controlled trials

**DOI:** 10.1097/MD.0000000000017099

**Published:** 2019-09-13

**Authors:** Jiawei Qin, Yi Zhang, Lijian Wu, Zexiang He, Jia Huang, Jing Tao, Lidian Chen

**Affiliations:** a College of Rehabilitation Medicine, Fujian University of Traditional Chinese Medicine, Fuzhou; b Department of Rehabilitation Medicine, Quanzhou First Hospital Affiliated to Fujian Medical University, Quanzhou; c Collaborative Innovation Center for Rehabilitation Technology, Fujian University of Traditional Chinese Medicine, Fuzhou, China.

**Keywords:** disability, low back pain, randomized controlled trial, rehabilitation, Tai Chi

## Abstract

**Background:**

This is the first systematic review evaluating and statistically synthesis the current studies regarding the effects of Tai Chi on pain and disability in patients with low back pain (LBP).

**Methods:**

Seven electronic databases including PubMed, EMBASE, Web of Science, Cochrane Library, China National Knowledge Infrastructure (CNKI), Wanfang and VIP information from inception to early March 2019 were searched. The Physiotherapy Evidence Database (PEDro) Scale was used to assess quality of all included randomized controlled trials (RCTs). The pooled effect size (weight mean difference, WMD) and 95% confidence interval (CI) were calculated to determine the effect of Tai Chi on pain and disability among LBP patients based on random effects model.

**Results:**

The aggregated results of the meta-analysis suggested that Tai Chi significantly decreased pain (WMD = −1.27, 95%CI −1.50 to −1.04, *P* < .00001, *I*^2^ = 74%) and improve function disability, Oswestry disability index (ODI) subitems: pain intensity (WMD = −1.70, 95% CI −2.63 to −0.76, *P* = .0004, *I*^2^ = 89%); personal care (WMD = −1.93, 95% CI −2.86 to −1.00, *P* < .0001, *I*^2^ = 90%); lifting (WMD = −1.69, 95% CI −2.22 to −1.15, *P* < .0001, *I*^2^ = 66%); walking (WMD = −2.05, 95% CI −3.05 to −1.06, *P* < .0001, *I*^2^ = 88%); standing (WMD = −1.70, 95% CI −2.51 to −0.89, *P* < .0001, *I*^2^ = 84%); sleeping (WMD = −2.98, 95% CI −3.73 to −2.22, *P* < .00001, *I*^2^ = 80%); social life (WMD = −2.06, 95% CI −2.77 to −1.35, *P* < 0.00001, *I*^2^ = 80%) and traveling (WMD = −2.20, 95% CI −3.21 to −1.19, *P* < .0001, *I*^2^ = 90%), Japanese Orthopedic Association (JOA) score (WMD = 7.22, 95% CI 5.59–8.86, *P* < .00001, *I*^2^ = 0%), Medical Outcomes Study Questionnaire Short Form 36 Health Survey (SF-36) physical functioning (WMD = 3.30, 95% CI 1.92–4.68, *P* < .00001), and Roland-Morris Disability Questionnaire (RMDQ) (WMD = −2.19, 95% CI −2.56 to −1.82, *P* < .00001).

**Conclusion:**

We drew a cautious conclusion that Tai Chi alone or as additional therapy with routine physical therapy may decrease pain and improve function disability for patients with LBP. Further trials are needed to be conducted with our suggestions mentioned in the systematic review.

## 1 Introduction

Low back pain (LBP) is a common symptom occurring in people of all age, typically located between the lower ribs and the buttock creases, sometimes accompanying by leg pain or lower limbs neurological symptoms.^[1]^ There are 7.3% worldwide population suffering activities limited caused by LBP at any one time, with the increasing trend in low to middle income countries.^[2]^ A recurrent rate is around 33% within 1 year since recovery from a last episode.^[3]^ So far, LBP is the leading cause of disability which result in growing burden to social and health systems.^[2]^ The global burden of diseases (GBD) reported that LBP was responsible for proximately 60 million years lived with disability in 2015.^[4]^ LBP is the most common reason leading to work-related day-off comparing with other occupational musculoskeletal disorders, resulting in 2.6 million visits to emergency a year in America.^[4]^

The specific source causing LBP usually could not be identified which was classified as non-specific low back pain (NSLBP).^[5]^ In contrast, there are a small portion of low back pain population caused by serious pathology abnormalities or disease (such as malignancy, vertebrae fracture, infection, spondylolisthesis, etc) that require careful identification and specific managements.^[6]^ Generally, NSLBP is multidimension problem including biophysical structure, psychological and social factors, leading negative effects to function, social participation, and financial prosperity.^[7]^ The altered spine stabilization function was detected among patients who suffered from LBP, and deep muscular stabilizer were considered to be associated with spine stability.^[8]^ Diaphragm, as an important deep muscular stabilizer, provided proper stability and appropriate motor control to the spine.^[9]^ A research showed that a reduced diaphragm thickness in athletes with LBP compared with healthy controls by rehabilitative ultrasound imaging.^[9]^ The psychological factors were related with development of LBP, including mood and emotions, cognitive function, depressive disorders.^[10,11]^ Patients with subacute LBP had higher Beck Depression Inventory (BDI) scores compared with healthy group.^[12,13]^ Furthermore, the patients with nonspecific acute and subacute LBP aging from 40 to 80 showed significantly higher depression scores by BDI scores by age distribution.^[14]^ A systematic review found that the person who ever experienced LBP had higher risk of recurrence, and people with chronic diseases or conditions (such as headache, diabetes, mental illness, smoking, heavy workload, etc) were more likely to suffer LBP.^[15]^ It is a pressing matter of the moment to identify cost-effective and specific strategies to relieve current and future burden.^[15]^

Many clinical guidelines^[16–19]^ present similar opinion about treatment of LBP, non-pharmacological therapy should be paid much more attention to deal with LBP rather than pharmacological therapy and surgery treatments during past 30 years. Guidelines recommend advice to maintain active, education, exercise therapy, and cognitive behavior therapy as first-line treatments, and spinal mobilization, massage, and acupuncture etc as second-line treatments.^[20]^ The characteristics of Tai Chi just have consistent principles with first-line treatment recommended by the clinical guidelines for LBP. Tai Chi as a low-moderate intensity, mind-body exercise originated from China, getting more and more popularity all-over the world.^[21]^ A research demonstrated that practicing Tai Chi had lower energy metabolism compared with general exercise, but similar health benefits in terms of aerobic fitness, resting energy expenditure, body composition, and self-perceived physical health.^[22]^ Tai Chi ranked top 3 most widely adopted complementary therapies in America from the national health interview survey.^[23]^ A previous reviews reported that Tai Chi was general safe exercise and unlikely to result in serious adverse events.^[24]^ There were limited reviews^[25,26]^ showing that Tai Chi could improve pain and disability significantly compared with waitlist, as these studies was conducted for several years already or including very limited relevant literatures. Chinese published studies were not included in some reviews^[25,27]^ as the reasons maybe languages barrier or limited search source.

As many new researches are continuing to be conducted and published, no review was done to evaluate the existing studies critically about Tai Chi treating LBP. This systematic review was conducted to assess the effectiveness of Tai Chi alone or as additional therapy in patients with LBP.

## 2 Methods

We had submitted the protocol to the International Prospective Register of Systematic Review before conducting this project (registration number CRD42018114648). We followed the Preferred Reporting Items for Systematic Review and Meta-analysis (PRISMA) to accomplish this project. We did not apply for ethical approval and patient consent since all analyses of this study were based on previous published literatures.

### 2.1 Data sources

The following electronic resources were searched from their inception time to March 2, 2019 which included PubMed, EMBASE, the Web of Science, Cochrane library, CNKI, Wanfang database, and VIP information. The languages of searched articles were not restricted. The literature search was executed around 2 key terms “Tai Chi” and “Low back pain.” For example, the following search strategy were used (tai ji OR Tai-ji OR Tai Chi OR chi, tai OR Tai Ji Quan OR Ji Quan, Tai OR Quan Tai Ji OR taiji OR Taijiquan OR T’ai chi OR Tai Chi Chuan OR shadowboxing) and (low back pain^[28]^ OR low back pains OR lumbago OR lower back pain OR lower back pains OR low back ache OR low back aches OR low backache OR low backaches OR vertebragenic pain syndrome OR vertebrogenic pain syndromes) in PubMed.

The reference lists of original and review articles were manual searched subsequently after completing the electronic searches. Under the guidance of the Cochrane Handbook, two researchers (JQ and YZ) searched all databases with the established strategy and screened the duplicate literatures individually using Endnote X8; the third investigator (JH) resolved disagreements between the initial 2 researchers if necessary.

### 2.2 Inclusion and study selection

Studies were included which met the following criteria: clinical randomized controlled trials (RCTs); included patients with a primary symptom of LBP; Tai Chi was the only intervention or in combination with other treatments; studies outcome measures should cover at least one of two essential assessments: pain, disability. To analysis the effects of Tai Chi for LBP, the following RCTs comparisons are eligible in this systematic review: Tai Chi versus control (waiting-list, unaltered lifestyle); Tai Chi + other treatment versus other treatment (other treatment is the same in both groups, such as, physical therapy, massage). Pain and disability were measured by visual analog scale (VAS), numerical pain rating scale (NPRS), Oswestry disability index (ODI), Japanese Orthopedic Association (JOA) score, or Roland-Morris Disability Questionnaire (RMDQ), etc.

Two reviewers (JQ and YZ) independently read through the titles and abstracts of the searched studies to exclude obvious irrelevant studies. The full text studies were then included according to the selection criteria. Detailed data were extracted by 1 reviewer (LW) in the prepared forms and the second reviewer (ZH) checked for the accuracy of the data. Disagreements were discussed between reviewers to reach a consensus.

### 2.3 Assessment of study quality

The methodological quality of included studies was assessed by 2 reviewers (JQ and YZ) using the Physiotherapy Evidence Database scale (PEDro). This scale contained 11 items with a maximum score of 10 points. The following information was assessed: random allocation; concealed allocation; baseline comparability; blind subjects; blind therapists; blind assessors; adequate follow-up; intention-to-treat analysis; between-group comparisons; point estimates; and variability. The score categorized <4 points as poor quality; 4 to 5 points was fair quality; 6 to 8 points was good quality; 9 to 10 was excellent quality. The RCTs which quality rating from “fair” to “excellent” was suitable for systematic review of physical therapy studies. Disagreements were consulted by obtaining the consensus of all reviewers.

### 2.4 Data extraction and synthesis

Two reviewers (LW and ZH) finished data extraction independently. The detailed data characteristic from extracted studies contained first author, year, location of study, published language, sample size, attrition rate, age of participants, course of disease, experimental and control interventions, frequency of Tai Chi exercise, duration, total sessions, Tai Chi style, outcomes and adverse effects (AE). The original author was contacted when the relevant information was not reported.

Review Manager software [RevMan 5.3 (The Nordic Cochrane Centre, The Cochrane Collaboration, Copenhagen, Denmark)] was used to conduct this meta-analysis. Statistical heterogeneity among the studies was assessed by a chi-square test and *I*^2^ value. The heterogeneity was quantified using the *I*^2^ index, where *I*^2^ > 25% indicated moderate heterogeneity; *I*^2^ > 50% substantial heterogeneity; and *I*^2^ > 75% considerable heterogeneity. When the *P* value of this test was <.1 and *I*^2^ value was >50%, a random effects model was used. Otherwise, a fixed effect model was carried out. We used the standardized mean difference (SMD) or the weight mean difference (WMD) and the 95% confidence interval (CI) to analyze the studies. Authors were contacted with by e-mail to obtain missing data for synthesis when possible. The standard deviations (SD) were estimated by using the formula suggested in the Cochrane Handbook for Systematic Reviews of Interventions when no response was available. We considered *P* < .05 as statistically significant. Sensitivity analysis was conducted to explore the source of heterogeneity and the stability of the result by excluding the inclusion of the studies one by one. Funnel plot asymmetry was employed to assess possible publication bias.

## 3 Results

### 3.1 Study selection

**Figure 1:**
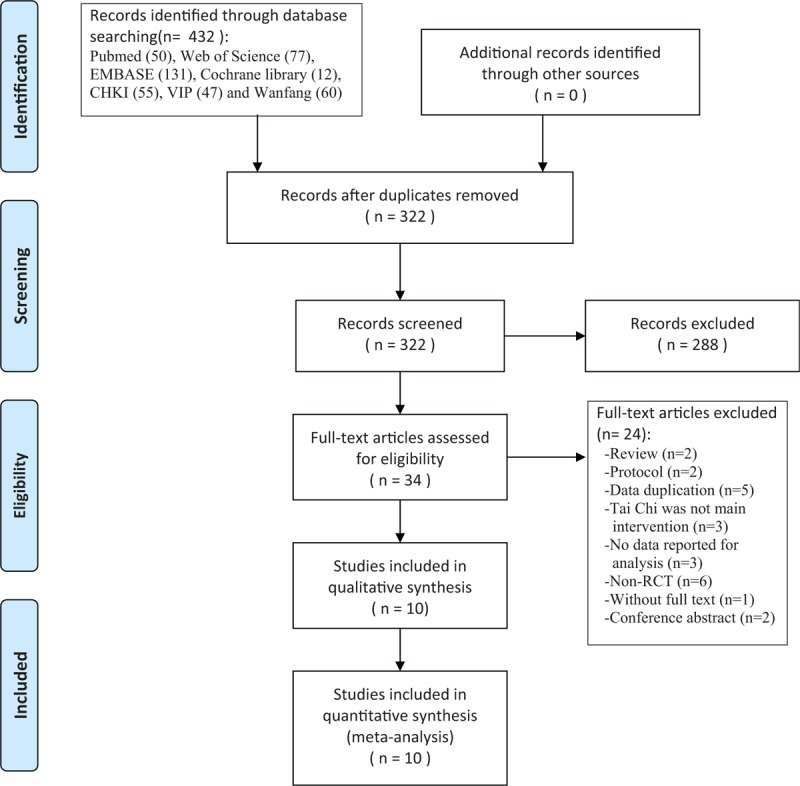
Flowchart of literature selection.

The detailed flowchart about the screening for eligible articles was displayed in Fig. 1. A total of 432 relevant records were identified through 7 English and Chinese electronic resource searches. After removing 110 duplicate articles, 322 articles remained to be screened for eligibility. Then 288 articles were excluded because they were irrelevant, 35 articles selected for a full text evaluation. Of these, 24 studies were excluded (reviews = 2, protocols = 2, data duplication = 5, Tai Chi was not main intervention = 3, no data reported for analysis = 3, non-randomized controlled trials = 6, without full text = 1, conference abstract = 2). Consequently, 10 RCTs were included into meta-analysis, 4 studies were published in English, and 6 in Chinese.

### 3.2 Study characteristics and methodological quality

A total of 10 studies involving 959 subjects (attrition rate ranging from 3.7% to 27.5%) with a mean age ranging from 32.6 ± 6.46 to 60.67 ± 2.58 years were included. These studies were conducted in China and Australia between 2008 and 2019. Across all the 10 RCTs, 5 studies experiment groups received Tai Chi alone, and their control groups received no treatment 29 to 33. The other 5 studies experiment groups received Tai Chi combined with other treatments (such as health education, massage, and routine physiotherapy) which were the same treatments conducted in control groups 34 to 38. For the Tai Chi intervention groups, half of them used Chen style Tai Chi 29,32,33,35,37, 1 practiced Tai Chi yun hand 38,1 practiced Tai Chi tui shou 30. The course of low back pain mentioned in 7 studies 29–31, 33, 35–37 was at least 3 months while other 3 studies did not report the course of low back pain. The treatment duration ranged from 2 weeks to 28 weeks with total sessions ranging from 12 to 168. The majority of the sessions lasted about 40 to 60 minutes, only 2 studies 30, 38 did not present detailed information on time of one session but did report the intervention program in detail. Professional or trained Tai Chi instructors taught participants or led the intervention in most included studies. The outcome measurement for pain were the VAS and NPRS, outcome measurement for disability were the ODI, followed by JOA score, only 1 study used SF-36 physical functioning 35 and RMDQ 31, respectively. Only 1 study 31 reported adverse effect during experiment. The main characteristics of all included studies were shown in Table 1.

**Table 1:**
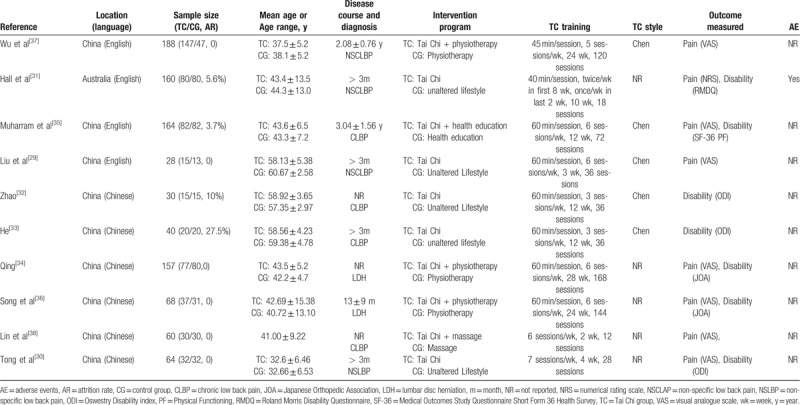
Summary of included studies.

### 3.3 Methodological quality

As shown in Table 2, methodological quality of all included 10 RCTs according to the PEDro scale were ranging from 4 to 7 points, which meant fair to good quality. Blinding of subjects and therapists were absent in all included RCTs. And 7 RCTs^[29,30,33–37]^ did not describe concealed allocation procedure in details, 7 RCTs^[30–34,36,38]^ did not perform assessors blinding, 6 trails^[30,32,33,35,37,38]^ rated the intention-to-treat negative, only 1 study^[33]^ showed >15% dropout. Other items were rated positive in all of the included RCTs.

**Table 2:**
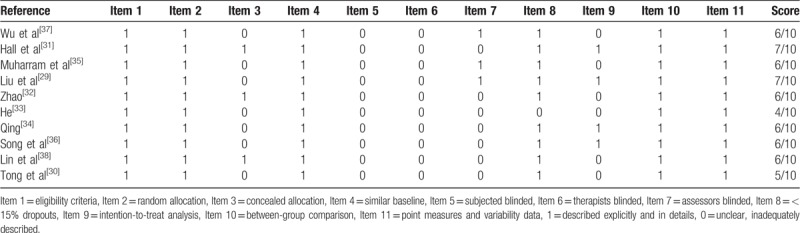
Quality assessment for eligible randomized controlled trials.

#### 3.3.1 Effect of Tai Chi in pain

**Figure 2:**
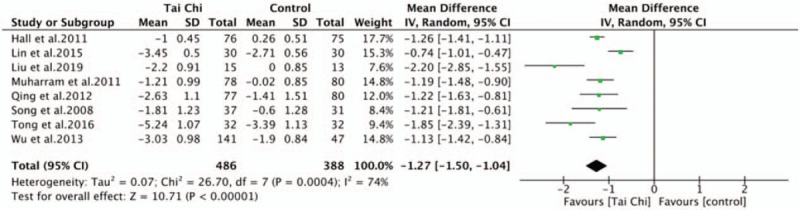
Forest plot of effect of Tai Chi for pain.

Meta-analysis of pain included 874 participants in 8 RCTs. The assessment tool for pain were different, 6 studies^[29–31,34,36,38]^ using 0 to 10 mm VAS or 0 to 10 NPRS, 2 studies^[35,37]^ using 0 to 100 mm VAS. We converted all these scales to a 0 to 10 points scale, the WMD and 95% CI were used calculated by using random-effect models. The aggregated results showed Tai Chi, alone or combined with other therapy, significantly reduced LBP pain level than control group (WMD, −1.27; 95% CI, −1.50 to −1.04; *P* < .00001; *I*^2^ = 74%; Fig. 2). Sensitivity analysis was conducted by removing these studies one by one since the heterogeneity was substantial with *I*^2^ = 74%, resulting in no significant changes which revealed that the pooled result was stable.

**Figure 3:**
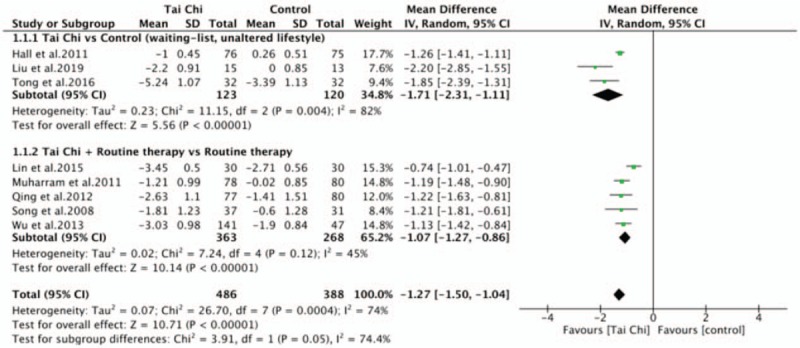
Forest plot of subgroup analysis of Tai Chi for pain.

A subgroup analysis was performed to explore differences between Tai Chi alone with Tai Chi combined with routine therapy. Tai Chi alone was superior to control group with waiting-list, unaltered lifestyle in pain intensity (WMD, −1.71; 95% CI, −2.31 to −1.11; *P* < .00001; *I*^2^ = 82%). Tai Chi combined with routine therapy (physiotherapy, massage, and health education) was superior to control group with the same routine therapy in pain intensity (WMD, −1.07; 95% CI, −1.27 to −0.86; *P* < .00001; *I*^2^ = 45%). Test for subgroup differences demonstrated no statistical difference (*P* = .05; *I*^2^ = 74.4%) (Fig. 3).

#### 3.3.2 Effect of Tai Chi in disability

**Figure 4:**
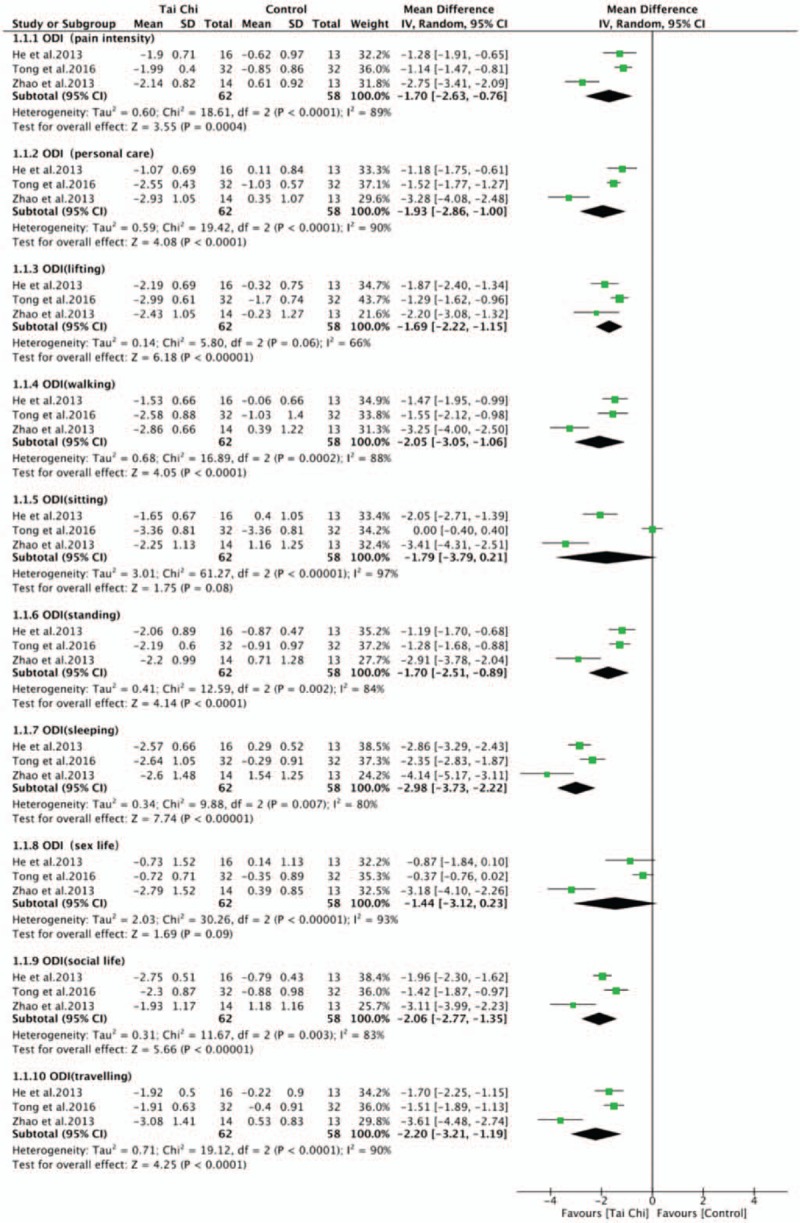
Forest plot of effect of Tai Chi for disability (ODI subitems). ODI = Oswestry disability index.

There were 7 RCTs^[30–36]^ measuring the disability changes by different assessment tools, 3 trials^[30,32,33]^ using ODI, 2 trials^[34,36]^ using JOA scores, 1 trial^[35]^ using SF-36 physical functioning, 1 trial^[31]^ using RMDQ. Higher scores indicated severer disability in ODI and RMDQ, while lower scores indicating severer disability in JOA and SF-36 physical functioning. The aggregated results of ODI subitems were assessed by 3 studies^[30,32,33]^ to compare the disability between Tai Chi group and control group, the results showing that Tai Chi group had more positive effect on these ODI subitems: pain intensity (WMD, –1.70; 95% CI, −2.63 to −0.76; *P* = .0004; *I*^2^ = 89%); personal care (WMD, −1.93; 95% CI, −2.86 to −1.00; *P* < .0001; *I*^2^ = 90%); lifting (WMD, –1.69; 95% CI, −2.22 to −1.15; *P* < .0001; *I*^2^ = 66%), walking (WMD, −2.05; 95% CI, −3.05 to −1.06; *P* < .0001; *I*^2^ = 88%), standing (WMD, −1.70; 95% CI, −2.51 to −0.89; *P* < .0001; *I*^2^ = 84%), sleeping (WMD, −2.98; 95% CI, −3.73 to −2.22; *P* < .00001; *I*^2^ = 80%), social life (WMD, −2.06; 95% CI, −2.77 to −1.35; *P* < .00001; *I*^2^ = 83%) and traveling (WMD, −2.20; 95% CI, −3.21 to −1.19; *P* < .0001; *I*^2^ = 90%), and no significant improvement on sitting (WMD, −1.79; 95% CI, −3.79 to 0.21; *P* = .08; *I*^2^ = 97%) and sex life (WMD, −1.44; 95% CI, −3.12 to −0.23; *P* = .09; *I*^2^ = 93%) (Fig. 4).

**Figure 5:**
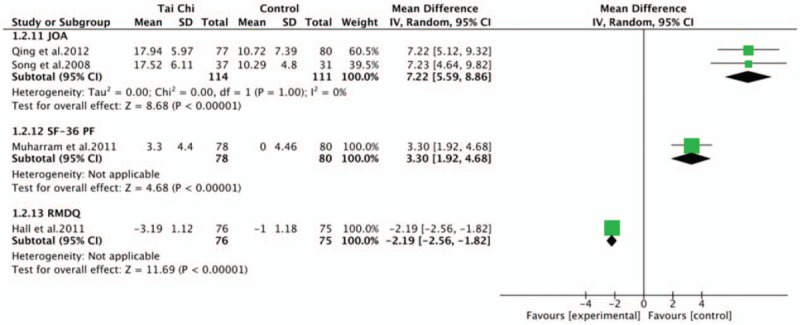
Forest plot of effect of Tai Chi for disability (JOA score, SF-36 PF, RMDQ). JOA = Japanese Orthopedic Association, RMDQ = Roland-Morris Disability Questionnaire, SF-36 = Medical Outcomes Study Questionnaire Short Form 36 Health Survey.

Two studies^[34,36]^ suggested that Tai Chi group outperformed the control group in terms of improving the JOA score for low back pain (WMD, 7.22; 95% CI, 5.59–8.86; *P* < .00001; *I*^2^ = 0%). Muharram et al^[35]^ considered Tai Chi group also had a significant improvements on physical functioning (WMD, 3.30; 95% CI, 1.92–4.68; *P* < .00001), as a section of The SF-36 Health Survey. Hall et al^[31]^ reported that Tai Chi group had a significant improvements on health status for low back pain (WMD, −2.19; 95% CI, −2.56 to −1.82; *P* < .00001) after 12 weeks intervention, as measured by RMDQ (Fig. 5).

### 3.4 Publication bias

**Figure 6:**
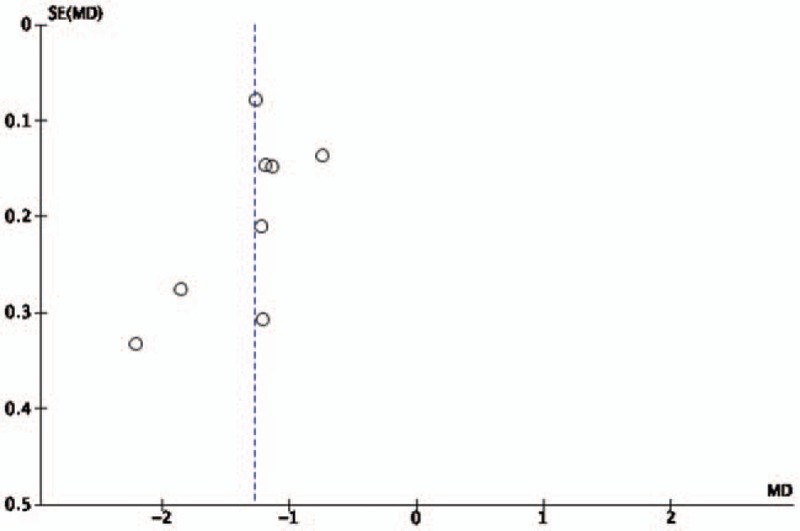
Funnel plot of Tai Chi for pain.

The funnel plot for pain was performed including 8 studies, showing small publication bias because of these spots were generally symmetric (Fig. 6).

### 3.5 Adverse events

Only one study^[31]^ reported adverse events that 3 participants had small increase in pain, which eliminated after 3 weeks, and 1 participant experienced an upper back pain which was alleviated by correcting the posture of the upper extremity.

## 4 Discussion

This systematic review and meta-analysis were conducted to evaluate the effect of Tai Chi alone or as additional therapy for LBP. The pooled estimates showed that Tai Chi could alleviate the pain level and improve functional disability for the patients with LBP. To the best of our knowledge, this is the first meta-analysis that assesses the efficacy of Tai Chi in pain and disability of LBP patients. The results of this systematic review are of a great importance for the patients with LBP who having different degrees of pain and disability which have negative influence on function and quality of life. Tai Chi could be implemented to those LBP patients as a safe, convenient, and inexpensive complementary treatment.

Previous review^[39]^ have evaluated the effect of traditional Chinese exercise including Tai Chi, baduanjin, qigong, etc on neck and back pain. The result of our review is consistent with its conclusion which just reported one large RCT that showing Tai Chi can decrease pain and improve function better than waitlist. Our review was also consistent with Kongs review,^[40]^ in which the aggregated result demonstrated that Tai Chi significantly reduced pain intensity of LBP patient. In contrast, Hall’ review^[41]^ found very low quality evidence that Tai Chi was more effective than waitlist for short-term LBP, and the difference was not statistically significant. It may due to the limited quantity of eligible included RCTs in Yuan's^[39]^ and Kong's^[26]^ reviews, and not including Chinese published researches in Hall's review,^[41]^ as well as variable Tai Chi style, treatment duration, frequency, and total sessions. We noticed that Tai Chi intervention parameter was substantial variable, intervention duration ranging from 2 to 28 weeks, total session ranging from 12 to 168 sessions. We found that longer intervention duration (such as 24 and 28 weeks) and more total sessions (such as144 and 168 sessions) had no significant improvement compared with shorter duration (such as 12 weeks) and less total sessions (such as 12 and 18 sessions) respectively in reduce pain intensity in LBP patients.

Tai Chi was an ancient exercise originating from China in the 13th century which including sequential slow and relaxed physical movements combined with deep breathing. The benefit mechanism of Tai Chi treating low back pain was based Chinese traditional medicine theory which principles were yin and yang and qi.^[27]^ Qi was considered as the tiny substance flowing in the human body, related with the normal function of human body and viscera. The person could maintain healthy if there was a balance between yin and yang, qi. Tai Chi training could resulting in a balance in yin and yang and improving the free-flowing of qi, leading better physical and mental health.^[27]^ Tai Chi was a complex therapy combined physical exercise and mind-body treatment that can alleviate pain, reduce stress, improve function, and quality of life.^[42]^ The other mediating factors were considered to be related with musculoskeletal strength improvement and level of physical activity.^[43]^ Practicing Tai Chi could improve lower limb strength and flexibility because of the consistent double-leg and single-leg squatting and weight shifting movements throughout the exercise.^[44]^ Improved lower limb function resulted in improving physical activities associated low back pain, such as sitting and standing, climbing stairs, and walking. The participants of Tai Chi practicing combined deep breathing with physical movements, may decrease muscle tension and pain.^[43]^ Besides, a review^[27]^ reported that poor pain related outcomes (i.e., pain intensity, disability) was consistent with higher pain-catastrophizing level. Pain-related catastrophizing was a negative cognitive response to pain. A study^[45]^ suggested that Tai Chi could reduce pain-related symptoms by having an influence on cognitive appraisal outcomes such as a reduction in catastrophizing.

Minimal clinically important difference (MCID), also named minimal clinically important change (MCIC), was considered as the minimal changes of symptoms which was clinically meaningful for patients.^[46]^ MCID could determine clinical relevance of outcomes, reflecting the effectiveness of intervention and patient response to the intervention. Previous studies^[47–51]^ concluded that improvement >1.2 NRS (0–10) points in patients of LBP should be a relevant change in pain intensity, and MCID values were 14.3 points of ODI, 2.5 points of JOA score and RMDQ, 3.5 points of SF-36 physical functioning subitems. The aggregated result of pain in this review was (WMD, −1.27; 95% CI, −1.50 to −1.04) that exceeded pain intensity MCID value 1.2 points slightly, suggesting the pooled result was clinical meaningful. However, the pain MCID value overlap the 95% CI of pooled result, the point estimated of 5 studies^[29–31,34,36]^ exceed 1.2 points while the points estimated of the other 3 studies^[35,37,38]^ were less than 1.2 points. Although the estimated effect of pain had substantial heterogeneity by *I*^2^ = 74%, the result had no significant changes after adopting sensitivity analysis by removing those studies one by one. Each ODI subitems were evaluated and pooled from 3 RCTs^[30,32,33]^ in this review, the weighting summary scores of all ODI subitems point estimated were 19.54 which far exceed ODI MCID value 14.3 points. The point estimated of JOA score^[34,36]^ was 7.22, far exceeding JOA MCID value. However, the pooled effect RMDQ^[31]^ and SF-36 physical functioning^[35]^ were a little less than corresponding MCID values, and only one study evaluated the disability by RMDQ and SF-36 physical functioning, respectively. Although these disabilities related outcome measure had statistically significant differences in tai chi group comparing with control group, the clinical meaning was still controversial. More large-sample and quality controlled strictly researches were needed to be conducted to identify the effectiveness of Tai Chi in disability for LBP patients.

## 5 Study limitations

Our meta-analysis also had several limitations. First, location and publication bias existed since 9 studies were conducted in China and only 1 study was conducted in Australia, 4 studies were published by English and 6 studies by Chinese. Second, although all included studies were RCTs, it was impossible to blind participants and Tai Chi instructors in all RCTs, concealed allocation and blinding outcome assessors could partly compensate for it, but only 3 studies reported assessor blinding and concealed allocation respectively. Third, most RCTs did not report long-term follow-up effect of Tai Chi on pain and disability of LBP patients. Fourth, the heterogeneity of pooled effect of pain was substantial although the results were relatively stable after conducting sensitivity analysis by removing studies one by one. Fifth, course of disease in the majority studies showed the participants suffered CLBP, the other studies did not report the detailed course of disease. Only 4 studies reported dropouts in which 1 study had a high dropout rate 27.5%, most studies were small sample size that one arm sample size was <40. It was suspicious that 5 studies conducted in China had non participants dropout. Sixth, Tai Chi style was not reported in a half of included studies, and frequency, treatment duration and total treatment sessions were also variable. In future, more larger and quality strictly controlled studies should be needed to explore the effectiveness of Tai Chi on LBP. Further studies can focus on different Tai Chi style, unique Tai Chi training frequency, and total sessions to validate the effects of Tai Chi for patients with LBP of different stages. The research of Tai Chi compared with other standard interventions (such as physical therapy, aerobic exercise, etc) in treating LBP can also be conducted. Such studies should follow the general accepted standard to conduct and report clinical trials, like consolidated standard of reporting trials statement (CONSORT).

## 6 Conclusion

Due to the different session, duration, frequency and style of the Tai Chi intervention, and different outcome assessment tools, and substantial heterogeneity of pooled results in this study, we drew a cautious conclusion that Tai Chi alone or as additional therapy with routine therapy may decrease pain intensity and improve function disability for patients with LBP. As a convenient, inexpensive, and nearly non-adverse event exercise, Tai Chi might be recommended for LBP patients, individually or integration with other conventional treatments. Finally, to establish positive effect of Tai Chi for LBP patients, further trials need to be conducted with specific Tai Chi style and dosage for different stages of LBP.

## Author contributions

**Conceptualization:** Jiawei Qin, Jing Tao, Lidian Chen.

**Data curation:** Yi Zhang, Lijian Wu, Zexiang He, Jia Huang.

**Formal analysis:** Jing Tao.

**Project administration:** Jiawei Qin.

**Software:** Yi Zhang.

**Supervision:** Jiawei Qin, Jia Huang, Jing Tao, Lidian Chen.

**Visualization:** Yi Zhang.

**Writing – original draft:** Jiawei Qin.

**Writing – review & editing:** Jing Tao, Lidian Chen.
